# Review of the Novel Investigational Antifungal Olorofim

**DOI:** 10.3390/jof6030122

**Published:** 2020-07-30

**Authors:** Nathan P. Wiederhold

**Affiliations:** Fungus Testing Laboratory, Department of Pathology and Laboratory Medicine, University of Texas Health Science Center at San Antonio, San Antonio, TX 78229, USA; wiederholdn@uthscsa.edu

**Keywords:** Olorofim, F901318, invasive mold infections, dihydroorotate dehydrogenase, *Aspergillus*, *Scedosporium*, in vitro susceptibility

## Abstract

The incidence of invasive fungal infections caused by molds and endemic fungi is increasing. There is also concern regarding increased rates of reduced susceptibility or frank resistance among *Aspergillus* and *Coccidioides* species, while *Scedosporium* species, *Lomentospora prolificans,* and *Fusarium* species are inherently less susceptible or intrinsically resistant to clinically available antifungals. Olorofim (formerly F901318) is the first member of the orotomide class of antifungals to be evaluated clinically for the treatment of invasive mold infections. This agent inhibits dihydroorotate dehydrogenase, a key enzyme in the biosynthesis of pyrimidines. Olorofim has activity against many molds and thermally dimorphic fungi, including species that are resistant to azoles and amphotericin B, but lacks activity against yeasts and the Mucorales. It is currently being developed for both oral and intravenous administration. Although published clinical outcome data have been limited to case reports to date, the results against invasive and refractory infections are promising. This review describes the mechanism of action of olorofim, its in vitro spectrum of activity, and what is currently known about its pharmacokinetic profile and clinical efficacy.

## 1. Introduction

Invasive infections caused by molds and thermally dimorphic fungi are of increasing clinical concern. This includes those caused by *Aspergillus, Scedosporium, Fusarium,* and *Coccidioides* species, among others. One of the most prevalent causes of mold infections in humans is represented by *Aspergillus* species. Surveillance studies have reported that invasive aspergillosis is a common cause of invasive infections in solid organ transplant recipients and hematopoietic stem cell transplant patients [1,2,3]. Chronic pulmonary aspergillosis is also a significant problem in patients with structural damage to the lungs, such as those who have had tuberculosis or sarcoidosis [4,5]. There is now increased recognition of azole resistance in *A. fumigatus* isolates, either due to clinical or environmental exposure to these agents [6,7,8,9,10,11], and studies have also reported increases in cryptic *Aspergillus* species, including those with reduced susceptibility to clinically available antifungal agents such as *A. lentulus, A. udagawae,* and *A. calidoustus* [12,13]. Other invasive mold infections that are associated with reduced susceptibility to clinically available antifungals and poor clinical outcomes include those caused by *Scedosporium* species and *Lomentospora* (formerly *Scedosporium*) *prolificans, Fusarium* species, as well as *Microascus* and *Scopulariopsis* species [14,15,16,17,18,19,20,21]. In addition, the incidence of infections caused by the endemic fungi *C. immitis* and *C. posadasii* has also been reported in parts of North America [22,23]. Although the clinical relevance is not completely understood, in vitro data have shown an increase in fluconazole resistance among *Coccidioides* isolates [24]. Thus, there is a significant medical need for the development of new antifungals.

**Figure 1:**
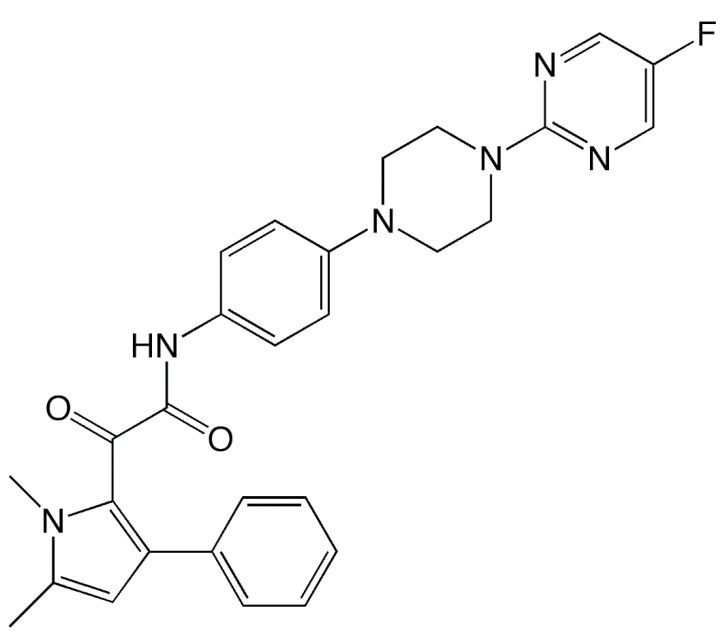
Structure of olorofim (F901318).

Olorofim (formerly F901318, F2G Ltd.; Figure 1) is the first member of a new class of antifungals, the orotomides, to reach clinical development. The mechanism of action of this novel agent is different than those of clinically available agents currently used to treat invasive fungal infections, which target either ergosterol within the fungal cell membrane or its biosynthesis (i.e., amphotericin B and azoles), or the biosynthesis of 1,3-β-d-glucan of the fungal cell wall (i.e., echinocandins). This review discusses the mechanism of action of olorofim, its spectrum of activity and effectiveness in animal models, and what is currently known about its clinical pharmacokinetic profile and efficacy in the treatment of invasive fungal infections. Key in vitro/in vivo findings from preclinical studies and clinical characteristics are provided in Table 1 and Table 2, respectively.

## 2. Mechanism of Action

Olorofim acts as a reversible inhibitor of the enzyme dihyroorotate dehydrogenase (DHODH), an oxidoreductase that catalyzes the fourth step in the de novo synthesis of pyrimidine [25]. The inhibition of pyrimidine biosynthesis results in the inhibition of the formation of uridine-5′-monophosphate (UMP) and uridine-5′-triphosphate (UTP). UMP and UTP are important for several cellular processes. UTP is required for the formation of UDP-sugars, which are substrates for 1,3-β-d-glucan synthase and chitin synthase (which are responsible for synthesis of the cell wall components 1,3-β-d-glucan and chitin, respectively) [26]. Thus, inhibition of pyrimidine synthesis by olorofim may affect the fungal cell wall and result in cell lysis [27]. An in vitro study reported that exposure of *A. fumigatus* hyphae to olorofim (0.1 μg/mL for 24 h) led to significant reductions in 1,3-β-d-glucan at the hyphal tips and at the periphery of the mycelium [28]. Interestingly, chitin content was significantly increased, which may be due to a compensatory mechanism that is known to occur with reduced 1,3-β-d-glucan levels following echinocandin exposure in different fungal species [29,30]. UTP is also important for the formation of the DNA/RNA pyrimidine derivatives cytosine, thymine, and uracil, as well as cell cycle regulation. Following 24 h of olorofim exposure, mitosis within *A. fumigatus* was halted, which is consistent with cell cycle arrest, and this was postulated to be due to a decrease in cellular levels of pyrimidine, leading to an inability to fully replicate DNA [28]. In addition to de novo synthesis, fungi are also able to acquire pyrimidine by scavenging from the environment. Although the addition of exogenous pyrimidine was shown to reverse the activity of olorofim in in vitro susceptibility assays, this only occurred at pyrimidine concentrations ≥5 mM, which is much higher than the concentration found within human serum (~15 μM) [25]. Thus, the amount of pyrimidine that can be scavenged by fungi from serum would not be sufficient to overcome the effects of olorofim.

Several other effects have been reported to occur following exposure of *A. fumigatus* to olorofim. Against conidia, olorofim has fungistatic activity through inhibition of germination, although isotropic growth continues [27]. Against germ tubes and vegetative hyphae, time-lapse live cell imaging demonstrated that the elongation of these forms immediately slowed following exposure to olorofim, and that with prolonged exposure (34 h), hyphal lysis occurred [27], which may be indicative of cell wall disorganization and weakening. These in vitro effects of olorofim against *A. fumigatus* appear to be time-dependent, as reduced viability (fungistatic activity) was observed against germ tubes and hyphae following 24 to 48 h of exposure, but cell death was observed after a more prolonged exposure (120 h) following hyphal swelling [27]. Interestingly, even with shorter exposures (24 h), hyphae appear to recover poorly, as regrowth has been shown to be significantly reduced or completely absent.

In addition to its effect on hyphal growth and viability, *A. fumigatus* exposure to olorofim has been shown to lead to increased hyphal septation, reduces the size of hyphal compartments, and increases in vacuolar volume [28]. The enlargement of vacuoles may be related to cell cycle arrest, as cytoplasmic volume may be an important trigger for the G1 cell cycle phase in which mRNA and proteins are synthesized in preparation for mitosis. Large vacuoles are formed under nutrient-limited conditions in order to decrease cytoplasmic volume, and this decreases the need for nutrients and protein synthesis [31]. Highly vacuolated compartments are believed to be arrested in the G1 cell cycle phase. It has been postulated that large vacuole formation may be a sign of the activation of autophagy in response to olorofim exposure [28]. It is currently unknown if the wide range of effects observed against *A. fumigatus* also occur against different fungal species when exposed to olorofim.

## 3. In Vitro Spectrum of Activity

The spectrum of activity of olorofim is unique in that it has activity against a variety of mold species and thermally dimorphic fungi, but lacks activity against yeasts, including *Candida* and *Cryptococcus*, as well as members of the order Mucorales [25,32,33,34,35,36]. In addition, the activity of olorofim against *Fusarium* appears to be species-specific [25,35]. This unique spectrum of activity of this agent and the orotomides in general is sequence-driven and predictive based on the phylogenetic analysis of DHODH [25]. *Candida, Cryptococcus,* and human DHODH are also mitochondrial class two DHODH, but are more distantly related to fungi that are inhibited by olorofim. In contrast, DHODH from the Mucorales are more closely related to class one DHODH enzymes, which are cytosolic [25]. It is known that other DHODH inhibitors also exhibit specificity based on inter-species variations in the hydrophobic channel of DHODH [25].

One of the most promising aspects of olorofim is its in vitro potency against fungi that are generally considered to have reduced susceptibility or frank resistance to currently available antifungal agents. This includes azole-resistant *Aspergillus* species, *Scedosporium* species and *L. prolificans,* which is pan-resistant to clinically available antifungals, and *Microascus* and *Scopulariopsis* species [25,32,34,35]. In vitro studies have reported that the activity of olorofim is unchanged against both azole-susceptible and azole-resistant *A. fumigatus* isolates, as well as cryptic species known to have reduced susceptibility to the azoles (e.g., *A. calidoustus, A. fumisynnematus, A. lentulus, A. pseudoviridinutans, A. tanneri, A. thermomutatus,* and *A. udagawae*, among others) [25,36,37,38,39,40]. Against azole susceptible wild-type *A. fumigatus,* olorofim minimum inhibitory concentrations (MICs) have ranged between 0.03 to 0.125 μg/mL, and this in vitro activity was also maintained by isolates harboring mutations within the *cyp51A* gene, including TR_34_/L98H and TR_46_/Y121F/T289A mutations [40]. Similar olorofim MIC ranges have also been reported against *A. flavus*, *A. nidulans, A. niger, A. tubinengensis,* and *A. terreus,* as well as the cryptic species *A. calidoustus* (section *Usti*)*, A. fumisynnematus, A. lentulus, A. pseudoviridinutans, A. thermomutatus,* and *A. udagawae* (section *Fumigati*)*, A. tanneri* (section *Aspergillus*)*,* and *A. alabamensis, A. citroniterreus,* and *A. hortai* (section *Terrei*) [25,36,37,38,39,40,41].

Against different *Scedosporium* species and *L. prolificans,* olorofim MICs have ranged between ≤0.008 to 0.5 μg/mL [32,34]. The MIC values for olorofim were lower than those observed for clinically available antifungal agents, including amphotericin B, itraconazole, voriconazole, and posaconazole, especially against *L. prolificans,* where the MIC ranges for the clinically available agents ranged from 0.5 μg/mL to more than 16 μg/mL, and most isolates demonstrated frank resistance to these clinically available antifungals. Olorofim has also been reported to have activity against *Microascus* and *Scopulariopsis* species (which frequently have reduced susceptibility to the azoles), as well as *Acremonium* species, *Paecilomyces variotii* (which has reduced susceptibility to voriconazole), *Purpureocillium lilacinum* (which is intrinsically resistant to amphotericin B), *Talaromyces marneffei,* the endemic and thermally dimorphic fungi *Blastomyces dermatitidis*, *Coccidioides* species, *Histoplasma capsulatum* [33,37], and *Madurella mycetomatis* (the main causative agent of eumycetoma) [42].

As previously noted, the in vitro activity of olorofim appears to be species-specific against *Fusarium* isolates. Very different susceptibility profiles have been reported against isolates belonging to the two most common species complexes associated with infections in humans, the *F. oxysporum* species complex and the *F. solani* species complex, with good activity against *F. oxysporum* (MIC range of 0.5–1 μg/mL) but limited to no activity against *F. solani* isolates (MIC range of 4 μg/mL to more than 8 μg/mL) at the 100% inhibition of growth endpoints used by the Clinical and Laboratory Standards Institute (CLSI) and the European Committee on Antimicrobial Susceptibility Testing (EUCAST) [35,43]. Against other *Fusarium* species, potent in vitro activity was observed against some but not all isolates, including *F. fujikuroi* (≤0.015–0.125 μg/mL), *F. proliferatum* (≤0.03 μg/mL to more than 8 μg/mL), and *F. verticillioides* (0.06–0.5 μg/mL); however, the number of isolates of these species tested was limited.

## 4. In Vivo Effectiveness

The in vitro activity that has been observed for olorofim against some mold species has also translated into in vivo efficacy in various animal models of invasive disease. In an immunocompromised murine model of invasive pulmonary aspergillosis, survival was significantly improved in mice infected with either an azole-susceptible *A. fumigatus* isolate or azole-resistant strains in which resistance was caused by TR_34_/L98H of G138C mutations in the *cyp51A* gene [44]. In addition, serum galactomannan was also significantly reduced in mice treated with olorofim, and both the survival results and reductions in serum galactomannan with olorofim were significantly greater than those treated with a humanized dose of posaconazole. Interestingly, survival and reductions in serum galactomannan where enhanced with more frequent dosing, and dose-fractionation experiments demonstrated time-dependent activity with trough (Cmin)/MIC being the pharmacokinetic/pharmacodynamic (PK/PD) parameter most closely associated with in vivo efficacy [44]. These observations are consistent with the time-dependent activity observed in vitro [27]. Similar results were also reported in a murine model of sinopulmonary aspergillosis secondary to *A. flavus* in which survival, changes in galactomannan, and histopathologic observations were the outcome measures [45]. In this study, reductions in galactomannan were exposure-dependent, and Cmin/MIC values (galactomannan index range associated with improved outcomes: 9–19) were predictive of both reductions in galactomannan and survival in mice treated with olorofim. Histopathologic clearance of fungi from lung tissue was also observed in several mice with olorofim treatment, and results were enhanced with more frequent dosing of this agent.

In experimental models of invasive aspergillosis in neutropenic mice and those with chronic granulomatous disease (*gp^−/−^ phox* mice) in which disease was caused by *A. fumigatus, A. nidulans,* or *A. tanneri*, treatment with olorofim resulted in improved survival and lead to reductions in galactomannan and fungal burden as measured by quantitative PCR under immunosuppressive conditions [39]. In addition, by histopathologic analysis, there were limited inflammatory foci and detectable fungal elements within different organs in mice treated with olorofim, and these effects occurred regardless of the azole susceptibility of the infecting *Aspergillus* isolate.

In vitro potency has also translated into in vivo efficacy in a murine model of central nervous system (CNS) coccidioidomycosis. In an established murine model in which arthroconidia of *C. immitis* were inoculated intracranially, treatment with olorofim significantly improved survival and reduced brain fungal burden, as measured by colony-forming units [33]. Both survival and reductions in brain fungal burden were enhanced when the olorofim dosing frequency was increased from twice daily to three times daily despite no changes in the overall daily doses. These results are in agreement with the time-dependent activity and Cmin/MIC as the PK/PD parameter associated with efficacy previously reported in murine models of aspergillosis.

## 5. Pharmacokinetics

The pharmacokinetics of olorofim have been reported from phase I clinical studies in healthy volunteers following both intravenous and oral administration. In an early study, olorofim was formulated in a β-hydroxypropyl-cyclodextrin vehicle due to its insolubility in water, and single ascending doses ranging from 0.25 to 4 mg/kg were administered by intravenous infusion over 4 h to healthy male volunteers [46]. The terminal half-life increased (30 h at the 4 mg/kg dose) as the doses increased, which is consistent with the decreased clearance also observed with higher doses. Olorofim was also rapidly distributed to the tissues, as demonstrated by a rapid plasma clearance and high volume of distribution (range: 2.89–3.49 L/kg), and less than 0.2% of the dose was recovered unchanged in the urine. The overall exposures as measured by the area under the concentration curve (AUC_0–inf._) increased in a dose proportional manner and ranged from 1.31–40.9 μg × hr/mL.

A subsequent multi-dose pharmacokinetic study was conducted in healthy male volunteers who were administered olorofim intravenously with infusion times ranging between 1 and 3 h [47]. A loading dose of 4 mg/kg twice daily was administered on day one followed by twice daily maintenance doses of 2.5 mg/kg on days two through five. Steady-state concentrations were reached within 24 h with this loading dose/maintenance dose strategy, and trough levels on day five ranged from between 1.3 to 2 μg/mL. The volume of distribution was approximately 3 L/kg, and the termination elimination half-life ranged from 20–30 h. Interestingly, secondary peaks were observed during the elimination phase, which may be indicative of enterohepatic recirculation of olorofim.

The pharmacokinetics of olorofim have also been evaluated with multiple oral dose administration. In this study, olorofim was formulated as an immediate release tablet containing 120 mg of active drug in a hydroxypropyl methylcellulose acetate succinate formulation [48]. Healthy male and female subjects were administered olorofim 360 mg once daily for 10 days under fasted conditions. Steady-state concentrations were achieved within three days, and trough levels were consistently measured between 1–2 μg/mL. As in the multiple intravenous dose study, secondary peaks were observed in the elimination phase, suggestive of enterohepatic recycling. Interestingly, higher exposures were observed in females (AUC_0-24_ 55.5 μg × hr/mL) than in males (AUC_0–24_ 35.6 μg × hr/mL), and this observation was linked to body weight (coefficient of determination (r^2^): 0.54). The bioavailability following oral administration has been reported to range between 45–82% in various animal species [49], and protein binding is >99% [50]. Although concentrations may reach therapeutic levels in the brain with repeated dosing, these concentrations are lower than those observed in the plasma, lungs, kidneys, and liver [25,49].

## 6. Adverse Effects and Drug Interactions

Currently, although little is known about the adverse effect profile of olorofim, inhibition of DHODH by this agent occurs in fungal-specific manner. In vitro, the potency of olorofim was significantly greater against recombinant *Aspergillus fumigatus* DHODH (half maximal inhibitory concentration [IC_50_]: 44 nM) compared to that against the recombinant human form of this enzyme (IC_50_ >100,000 nM; >2200-fold difference) [25]. In phase I pharmacokinetic studies, olorofim was well tolerated when administered intravenously or orally. In the multiple dose pharmacokinetic study in healthy male volunteers, no significant changes in vital signs or laboratory values were reported, nor were cardiovascular changes observed by echocardiogram (ECG) [47]. Infusion-related reactions (infusion site pain and phlebitis, 44% and 39%, respectively) and dizziness (67%) were the most commonly reported adverse events in the olorofim groups, and were observed at higher rates than in healthy volunteers who received placebo (17% for each adverse event in this group). The duration of infusion did not appear to impact the severity or frequency of these events. Olorofim was also well tolerated in the phase I study in which healthy volunteers were administered once daily oral doses for 10 days [48]. No severe or serious adverse effects were reported. Two subjects experienced mild increases in alanine aminotransferase levels, one subject experienced both nausea and diarrhea, and one had dizziness. No other significant changes in vital signs, laboratory tests, or ECG findings were observed.

Olorofim is metabolized by several cytochrome P450 isoenzymes, and thus drug–drug interactions are a concern [51]. Olorofim is not an inducer of CYP450 isoenzymes but has been shown to be a weak inhibitor of CYP3A4. In an open-label, healthy volunteer study, increases in midazolam concentrations were observed when dosed on day seven compared to when this benzodiazepine was administered on day one prior to the start of a seven day course of olorofim (mean midazolam concentration of 1.27 μg/mL on day one increased to 1.65 μg/mL on day seven) [52]. Additional studies and clinical observations will further elucidate the drug–drug interaction potential for olorofim. This may be clinically relevant given the drug–drug interaction profiles of numerous medications that are used in patients at risk for opportunistic invasive mold infections.

## 7. Clinical Outcomes

Olorofim is currently in an open-label, single-arm phase IIb clinical study for the treatment of invasive mold infections in patients with limited treatment options. This includes aspergillosis refractory or intolerant to clinically available antifungals, and infections due to *Scedosporium* species, *Lomentospora prolificans*, *Scopulariopsis* species, and other resistant fungi in patients without suitable alternative treatment options (ClinicalTrials.gov identifier: NCT03583164). In November 2019, olorofim was granted breakthrough drug therapy designation by the U.S. Food and Drug Administration (FDA). Subsequently, it was granted orphan drug designation (ODD) for the treatment of invasive aspergillosis and *Lomentospora/Scedosporium* infections (March 2020) and coccidioidomycosis (June 2020), and most recently as a qualified infectious disease product (QIDP; June 2020) for the treatment of the above-mentioned invasive fungal infections, as well as invasive fusariosis.

Currently, there are few published clinical data demonstrating the effectiveness of olorofim against invasive fungal infections, as most available data come from case reports in abstract form. However, these results are promising. Two such case reports have reported improved outcomes in patients with invasive infections due to *Lomentospora prolificans*. One involved a 56-year-old female status post-HyperCVAD cycle1-b for T-cell acute lymphoblastic leukemia who developed disseminated infection due to this pathogen, which involved the bloodstream, eyes (endophthalmitis), L4/5 vertebrae of the spine, lungs, and aortic valve [53]. The patient had failed therapy with voriconazole combined with terbinafine as well as surgical debulking of the spine. Olorofim monotherapy was started 11 months into the infection (loading dose of 180 mg followed by 60 mg twice daily, and later increased to 90 mg twice daily). Within six months, improvement was observed as demonstrated by serial positron emission tomography PET scans showing uptake at the aortic root and lumbar spine, weight gain and vision stabilization, and the patient received a year of olorofim therapy without adverse effects.

In the second case, a 49-year-old woman developed lomentosporiosis of the right breast implant with spread to the soft tissues, ribs and sternum, which failed treatment with implant removal, repeated debridement, hyperbaric oxygen, and multiple antifungals, including voriconazole terbinafine, miltefosine, posaconazole, and anidulafungin [54]. She started treatment with olorofim 60 mg twice daily, which was subsequently increased to 90 mg and finally 120 mg twice daily and a total olorofim duration of 322 days, which was well tolerated. Following the initiation of olorofim treatment, gradual healing of the surgical site and subsequent wound closure was achieved.

Olorofim has also been reported to result in a successful outcome in a patient with disseminated coccidioidomycosis with severe pulmonary disease and concurrent meningitis [55]. In this case, a 45-year-old male with insulin-dependent diabetes mellitus had been treated without success with fluconazole, voriconazole, and itraconazole. Due to continued deterioration, he was switched to liposomal amphotericin B plus posaconazole. Due to significant hypokalemia, the patient was switched to combination therapy with posaconazole and micafungin but without improvement. Eight months after the initiation of therapy, he was switched to posaconazole plus olorofim, administered at 120 mg twice daily. The patient reported rapid improvement of cough and malaise within a week, and after three months of combination therapy returned to his normal activity level without the need for supplemental oxygen. Improvement of in pulmonary multifocal infiltrates was observed by repeated computed tomography CT, and the *Coccidioides* complement fixation titer had decreased from 1:128 to 1:32 within five months. Although the results of these three cases are impressive, additional clinical results from the phase IIb open-label study as well as subsequent clinical trials are needed in order to truly judge the clinical efficacy of olorofim for the treatment of these invasive fungal infections.

The cases described above are currently available only in abstract form and do not detail how the organisms causing the infections were determined. One of the limitations of olorofim is its lack of activity against yeasts and certain filamentous fungi (i.e., the Mucorales). Thus, the use of this agent may be limited until the cause of the invasive fungal infection has been identified.

## 8. Conclusions

Olorofim is a promising investigational agent currently in clinical studies for the treatment of invasive mold infections and thermally dimorphic fungi that are refractory or resistant to clinically available antifungals. Its mechanism of action against pyrimidine biosynthesis is novel, and thus it is not affected by mechanisms that cause resistance to clinically available antifungals, including the azoles and amphotericin B. A major limitation is its lack of activity against yeasts, including *Candida* and *Cryptococcus* species, as well as members of the order Mucorales. Thus, there should be a strong level of evidence that infections are not caused by these fungi before olorofim is initiated. Although clinical outcome data are limited to date, the preliminary results that are available for the treatment of invasive infections caused by resistant organisms or those that are refractory to previous therapies are promising.

## Figures and Tables

**Table 1:** Key in vitro and in vivo features of olorofim (F901318).

Feature	Description
Mechanism of action	Targets pyrimidine biosynthesis through inhibition of dihydroorotate dehydrogenase
Spectrum of in vitro activity	*Aspergillus* spp., including azole resistant isolates *Scedosporium* spp., and *Lomentospora prolificans* Variable and species-specific vs. *Fusarium* Endemic fungi (*Blastomyces, Coccidioides, Histoplasma*) *Microascus/Scoulariopsis* spp. *Penicillium, Paecilomyces, Purpureocillium, Talaromyces* *Madurella* No activity against yeasts or the Mucorales
Activity in in vivo models	Improvements in survival and histopathology, reductions in galactomannan in a neutropenic murine model of pulmonary aspergillosis due to *A. fumigatus* (wild-type and isolates harboring mutations in *cyp51A* (TR_34_/L98H and G138C)) Improvements in survival and histopathology, and reductions in galactomannan in murine sinopulmonary model of invasive aspergillosis secondary to *A. flavus* Improvements in survival and histopathology, and reductions in galactomannan and tissue fungal burden in a CGD murine model of invasive aspergillosis secondary to *A. fumigatus, A. nidulans,* and *A. tanneri* Improvements in survival and reductions in brain tissue fungal burden in murine model of CNS coccidioidomycosis

CGD—chronic granulomatous disease; CNS—central nervous system.

**Table 2:** Clinical characteristics of olorofim (F901318).

Clinical Characteristic	Description
Pharmacokinetic parameters	Large volume of distribution (~3 L/kg); some CNS distribution High plasma protein binding (>99%) Half-life approximately 20–30 h Bioavailability >45% Pharmacokinetic/pharmacodynamic parameter Cmin/MIC
Clinical status	Currently in Phase IIb clinical study Case reports describe success against disseminated lomentosporiosis and coccidioidomycosis
Adverse effects/drug–drug interactions	Infusion site pain and phlebitis with intravenous infusion Dizziness Weak inhibitor of CYP3A4 Metabolized by multiple CYP450 isoenzymes

CNS—central nervous system; Cmin—trough; CYP—cytochrome P450.
